# Targeted Inactivation of Snail Family EMT Regulatory Factors by a Co(III)-Ebox Conjugate

**DOI:** 10.1371/journal.pone.0032318

**Published:** 2012-02-29

**Authors:** Allison S. Harney, Thomas J. Meade, Carole LaBonne

**Affiliations:** 1 Department of Molecular Biosciences, Northwestern University, Evanston, Illinois, United States of America; 2 Departments of Chemistry, Neurobiology, and Radiology, Northwestern University, Evanston, Illinois, United States of America; 3 Robert H. Lurie Comprehensive Cancer Center, Northwestern University, Evanston, Illinois, United States of America; Radboud University Nijmegen, The Netherlands

## Abstract

Snail family proteins are core EMT (epithelial-mesenchymal transition) regulatory factors that play essential roles in both development and disease processes and have been associated with metastasis in carcinomas. Snail factors are required for the formation of neural crest stem cells in most vertebrate embryos, as well as for the migratory invasive behavior of these cells. Snail factors have recently been linked to the formation of cancer stem cells, and expression of Snail proteins may be associated with tumor recurrence and resistance to chemotherapy and radiotherapy. We report that Co(III)-Ebox is a potent inhibitor of Snail- mediated transcriptional repression in breast cancer cells and in the neural crest of *Xenopus*. We further show that the activity of Co(III)-Ebox can be modulated by temperature, increasing the utility of this conjugate as a Snail inhibitor in model organisms. We exploit this feature to further delineate the requirements for Snail function during neural crest development, showing that in addition to the roles that Snail factors play in neural crest precursor formation and neural crest EMT/migration, inhibition of Snail function after the onset of neural crest migration leads to a loss of neural crest derived melanocytes. Co(III)-Ebox-mediated inhibition therefore provides a powerful tool for analysing the function of these core EMT factors with unparalleled temporal resolution. Moreover, the potency of Co(III)-Ebox as a Snail inhibitor in breast cancer cells suggests its potential as a therapeutic inhibitor of tumor progression and metastasis.

## Introduction

Snail family transcription factors, consisting of Snai1 (also known as Snail) Snai2, and Snai3 (also known as Slug and SMUC respectively), are a family of zinc finger transcriptional repressors that are key regulators of epithelial-mesenchymal transitions (EMTs) during embryonic development, and of metastasis in epithelial-derived carcinomas. The plastic interconversion of epithelial cells to mesenchymal cells allows cellular remodelling during wound healing, tissue regeneration in differentiated tissues in the adult, [1], [2], [3], [4] and is essential for numerous developmental processes, including gastrulation, neural crest cell migration, palatal fusion, and mammary branching morphogenesis [5], [6], [7], [8]. In *Xenopus*, *Snai2* and *Snai1* are among the earliest factors expressed in response to neural crest inducing signals. They play essential roles in both the establishment of the multipotent precursor population and for the subsequent EMT/migration of definitive neural crest cells [7], [9]_ENREF_9. Neural crest cells have emerged as an excellent model system for understanding the function and regulation of Snail proteins in both normal and pathological contexts.

Snail proteins, together with other core EMT/neural crest regulatory factors, are implicated in epithelial plasticity and EMT-like processes during tumor progression [10], [11]. *Snai1* expression, and features of EMT have been observed in breast, prostate, lung, ovarian, melanoma, colon and esophageal cancers [12], [13], [14], [15], [16], [17], [18]. Introduction of Snail factors into epithelial tumors results in cells that can disseminate from the primary tumor, are resistant to apoptosis, radiotherapy and chemotherapy, evade immune recognition, and exhibit markers of stem cells [19], [20], [21], [22]. Mechanistically, Snail proteins repress the expression of E-cadherin and components of adherens junctions, and *Snail* expression has been shown to correlate with tumor malignancy [17], [23], [24].

Snail factors are attractive targets for the development of pharmaceutical agents. Blocking Snail protein function has the potential to prevent tumor cell metastasis by interfering with processes such as EMT, cytoskeletal remodelling, cell migration and invasion. Moreover, the recent link between Snail and cancer stem cells [19], [25], [26] suggests that inhibitory agents could prove to be potent inhibitors of tumor recurrence. In addition to their potential as anti-cancer therapeutics, inhibitors of Snail function would be powerful tools that could facilitate the study of these transcriptional regulatory factors in model cell types such as the neural crest, and in other developmental processes.

**Figure 1:**
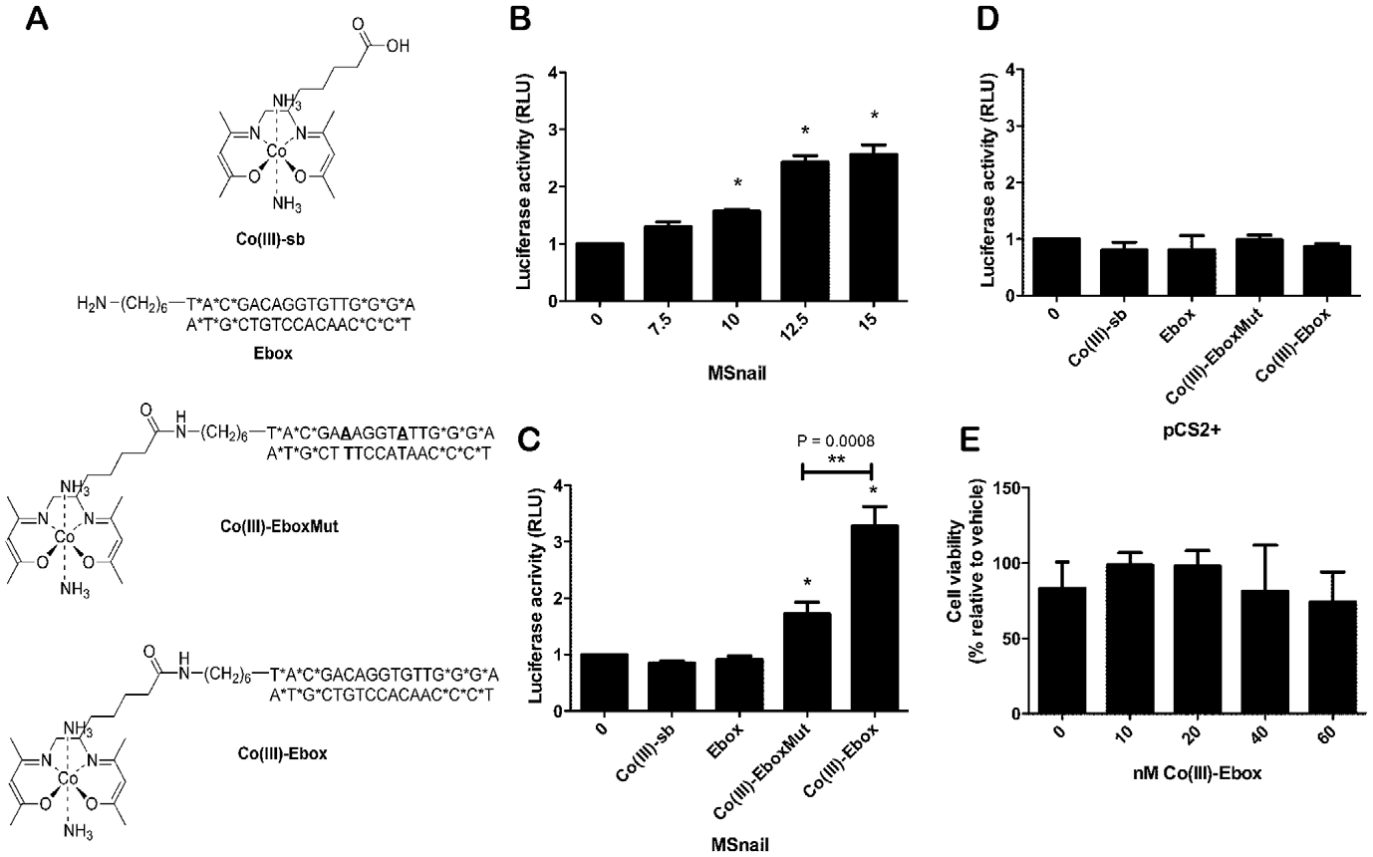
Co(III)-Ebox alleviates murine Snai1-mediated *E-cadherin* promoter repression. (A) Chemical structures; Co(III) Schiff base complex, Co(III)-sb; Ebox duplex oligonucleotide, Ebox; Co(III)-DNA conjugate with 2-base pair substitution in the Ebox region; Co(III)-EboxMut; Co(III)-DNA conjugate targeted to Snail factors, Co(III)-Ebox. (B) *E-cadherin* reporter gene activity in MCF7 cells expressing MSnail treated with Co(III)-Ebox from 0 to 15 nM. Data are represented as means ± s.e.m., n = 4. T-tests determined statistical significance from controls of 10 nM (P = 0.0034), 12.5 nM (P = 0.0060) and 15 nM (P = 0.0109) (*). (C) *E-cadherin* reporter gene activity in MCF7 cells expressing MSnail treated with 15 nM Co(III)-sb, Ebox, Co(III)-EboxMut or Co(III)-Ebox. Data are represented as means ± s.e.m., n = 4. T-tests determined statistical significance from controls of Co(III)-EboxMut (P = 0.0214) and Co(III)-Ebox (P = 0.0035) (*). Co(III)-Ebox to be significantly different from Co(III)-EboxMut (P = 0.0008) (**). (D) *E-cadherin* reporter gene activity in MCF7 cells transfected with pCS2+ (vector) treated with 15 nM Co(III)-sb, Ebox, Co(III)-EboxMut or Co(III)-Ebox. Data are represented as means ± s.e.m., n = 4. T-tests determined no statistical significance between the means. (E) MCF7 cell viability after treatment with Co(III)-Ebox at indicated concentrations after 24 h. Data are represented as means ± s.e.m., n = 3. T-tests determined no statistical significance between the means.

Co(III) Schiff base complexes can be used as selective inhibitors of transcription factors and enzymes through a rational design of the conjugated oligonucletide or peptide (Figure 1A) [27], [28], [29], [30]. Here, we report that a Co(III)-DNA conjugate, Co(III)-Ebox, is a potent inhibitor of Snail-mediated repression and EMT in the neural crest during embryonic development, and in breast cancer cells. In breast cancer cells, Co(III)-Ebox selectively inhibits Snail-mediated repression of the *E-cadherin* promoter in a dose dependent manner. *Xenopus* embryos treated with this agent display defects in the induction and/or migration of neural crest cells, depending upon the timing of administration. We find that inhibitory effects of this compound can be modulated with temperature, and we exploit this feature to further investigate the temporal requirements for Snail family factors in the neural crest.

Here, we show that the inhibition of Snail function after the onset of neural crest migration leads to a loss of neural crest derived melanocytes, further highlighting the multiple essential roles that Snail proteins play in the development of neural crest cells. The temporal control of Snail function afforded by Co(III)-Ebox provides a powerful new tool for dissecting the role these proteins play in cellular and developmental processes with unprecedented temporal resolution. The robust and specific inhibition of Snail function by Co(III)-Ebox in breast cancer cells suggests that this reagent holds considerable therapeutic potential as an inhibitor of tumor progression and metastasis.

## Results

### Inhibition of Snail DNA-binding and transcriptional repression in breast cancer cells

We examined the ability of Co(III)-Ebox to selectively block Snail-mediated transcriptional repression in tumor-derived cells. In initial experiments we exploited the fact that MCF7 breast cancer cells lack endogenous nuclear Snai1 (Figure S1) and that introduction of *Snai1* into these cells has been shown to repress *E-cadherin* luciferase reporter activity (Figure S2) [31], allowing for a clear link between the effects of the agent and the presence of its target. The effects of treatment with Co(III)-Ebox were compared to control treatment with vehicle, Co(III)-sb, Ebox oligonucleotide or Co(III)-EboxMut (Figure 1A). Co(III)-sb and Ebox were used to assess the effects of the Co(III) Schiff base or the Ebox oligonucleotide individually. Co(III)-EboxMut is a form of the conjugate in which the Ebox sequence has been mutated to diminish Snail protein binding, and was used to further evaluate the specificity of the binding interaction (Figure 1A, Figure S3).

MCF7 cells transfected with murine Snai1 (MSnail) and the luciferase reporter show a dose-dependent increase in *E-cadherin* promoter activity following treatment with Co(III)-Ebox. Significant changes in reporter gene activity were observed with 10 nM Co(III)-Ebox (P = 0.0034). Maximal effects were achieved by treatment with 15 nM Co(III)-Ebox (P = 0.0109, Figure 1B). Administration of Co(III)-Ebox significantly increased *E-cadherin* reporter activity while controls Co(III)-sb, Ebox or, Co(III)-EboxMut had reduced effects (1.7±0.21 fold compared to 3.3±0.35 fold change relative to vector control P = 0.0008) (Figure 1C). Off-target effects due to reaction of the compound with proteins from other pathways appear to make no significant contribution to the changes in luciferase activity, as the change in *E-cadherin* promoter activity was dependent on the presence of transfected MSnail. These data provide strong evidence that the effects of Co(III)-Ebox on *E-cadherin* repression are a consequence of its ability to inactivate Snail.

We previously demonstrated that Co(III)-Ebox selectively targets Ebox-binding zinc finger transcription factors [27]. In that study we found that Co(III)-Ebox does not inhibit either Ebox-binding transcription factors that lack zinc finger domains, or zinc finger containing proteins that do not bind Ebox consensus sequences [27]. These dual requirements are due to the two-part targeting mechanism utilized by Co(III)-Ebox. Proteins are first selectively targeted via their ability to bind the Ebox-containing oligonucleotide, and then irreversibly inactivated through interaction of the Co(III) Schiff base complex with histidine residues in the zinc finger region [27]. Consequently, the Ebox binding bHLH transcription factor MitF fails to be inhibited by concentrations of Co(III)-Ebox 100 fold higher than those that inhibit XSnai2 (Figure S4). Furthermore, treatment of MCF7 cells with Co(III)-Ebox had no impact on cell viability at the concentrations utilized for Snail inhibition (Figure 1E). No significant changes in viability were observed following treatment with concentrations of Co(III)-Ebox up to 60 nM (4 fold the working concentration).

**Figure 2:**
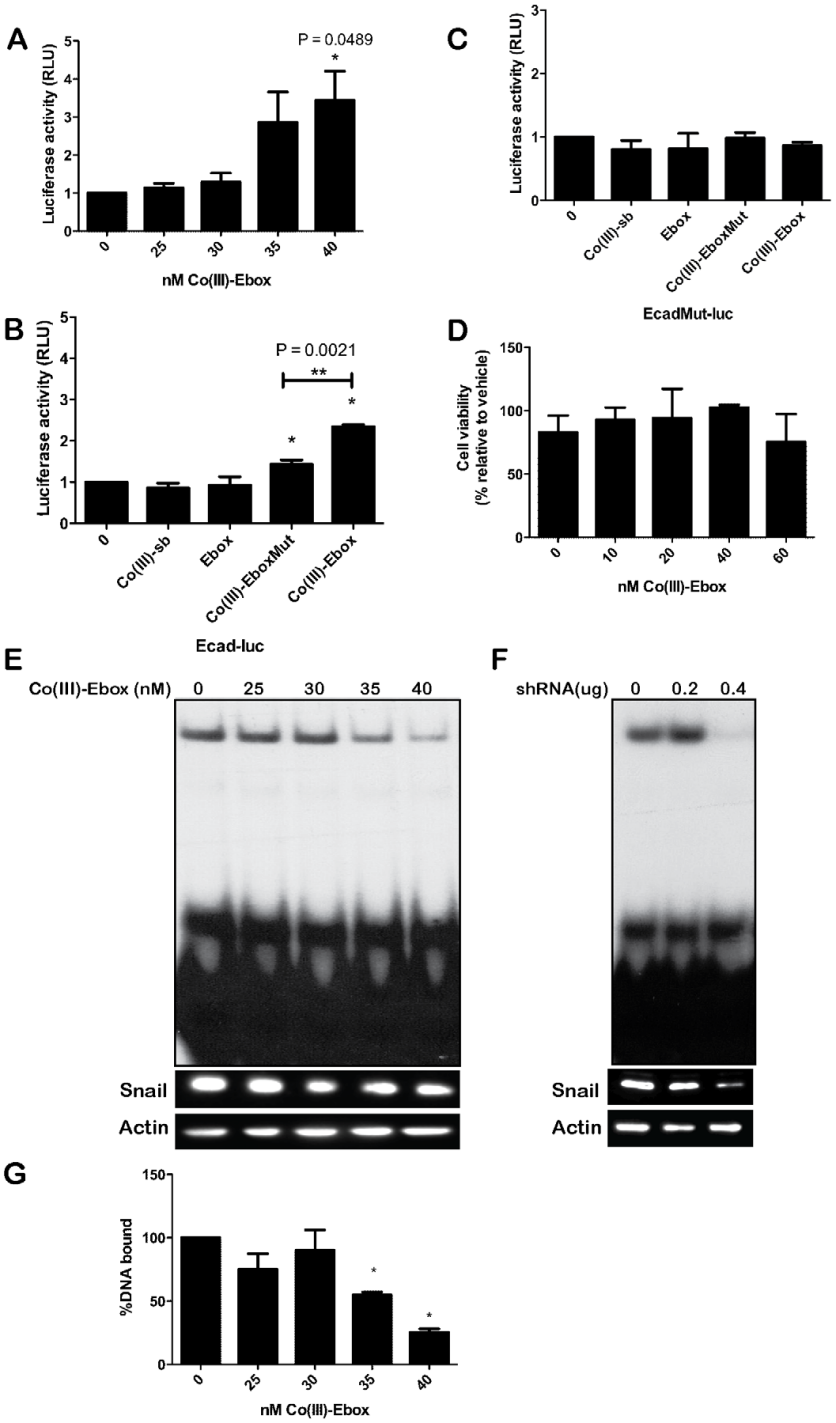
Co(III)-Ebox binds target proteins to alleviate transcriptional repression in metastatic MDA-MB-231 cells. (A) *E-cadherin* reporter gene activity in MDA-MB-231 cells treated with Co(III)-Ebox from 0 to 40 nM. Data are represented as means ± s.e.m., n = 4. T-tests determined statistical significance from controls of 40 nM (P = 0.0489) (*). (B) *E-cadherin* reporter gene activity in MDA-MB-231 cells treated with 35 nM Co(III)-sb, Ebox, Co(III)-EboxMut or Co(III)-Ebox. Data are represented as means ± s.e.m., n = 4. T-tests determined statistical significance from controls of Co(III)-EboxMut (P = 0.0290) and Co(III)-Ebox (P = 0.0001) (*). Co(III)-Ebox to be significantly different from Co(III)-EboxMut (P = 0.0008) (**). (C) Luciferase reporter gene activity in MDA-MB-231 cells with EcadMut-luc reporter treated with 35 nM Co(III)-sb, Ebox, Co(III)-EboxMut or Co(III)-Ebox. Data are represented as means ± s.e.m., n = 4. T-tests determined no statistical significance between the means. (D) MDA-MB-231 cell viability after treatment with Co(III)-Ebox at indicated concentrations after 24 h. Data are represented as means ± s.e.m., n = 3. T-tests determined no statistical significance between the means. (E) Inhibition of Snail DNA binding in MDA-MB-231 cells after 24 h with 0 to 40 nM Co(III)-Ebox. A representative EMSA of triplicate samples is shown with Western blots of Snai1 and Actin. (F) A representative EMSA of MDA-MB-231 cells treated with shRNA targeting *Snai1* with Western blots of Snai1 and Actin. (G) Quantification of the percentage of Slug probe bound to Snai1 in (E) by using a STORM 680 phosphoimager. Data are represented as the means ± s.e.m., n = 3.

Experiments in MCF7 cells demonstrated the ability of the Co(III)-Ebox conjugate to inhibit exogenously provided Snail protein. We next wished to evaluate the effectiveness of Co(III)-Ebox in blocking the function of endogenous Snail proteins. MDA-MB-231 cells are metastatic breast cancer cells that express multiple zinc finger transcriptional repressors, including *Snai1* (Figure S1), *Snai2*, *Zeb1* and *Zeb2*, all of which can bind Eboxes in the *E-cadherin* promoter and regulate EMT [32], [33], [34]. We have shown that DNA binding by Snai1, Snai2 and Zeb2 is inhibited by Co(III)-Ebox in vitro [27]. MDA-MB-231 cells therefore make an excellent model in which to assess ability of Co(III)-Ebox to inactivate the function of multiple distinct endogenous targets and alleviate the transcriptional repression of the *E-cadherin* promoter observed in these cells. We found that treatment of MDA-MB-231 cells expressing an *E-cadherin* reporter with Co(III)-Ebox led to a significant increase in promoter activity, with the greatest effects achieved at a dose of 40 nM Co(III)-Ebox (fold change 3.4±0.7, Figure 2A). No significant changes in *E-cadherin* promoter activity were noted following treatment with Co(III)-sb, or Ebox alone. As observed in MCF7 cells, the activity of Co(III)-EboxMut was significantly diminished compared to Co(III)-Ebox (P = 0.0021, Figure 2B). In previous studies, potential E-box independent effects on *E-cadherin* promoter regulation have been assessed using a control reporter in which two base-pair substitutions have been introduced into each of the three Eboxes (EcadMut-luc) resulting in decreased ability of Snai1, Snai2, Zeb1 and Zeb2 to bind these sites (Figure S5) [35]. Consistent with the observed effects on *E-cadherin* promoter activity being a consequence of regulation by endogenous E-box binding transcription factors, treatment with Co(III)-Ebox, (or controls Ebox, Co(III)-EboxMut or Co(III)-Ebox) had no effect on EcadMut promoter activity (Figure 2C). Cell viability was unaffected by treatment with Co(III)-Ebox (Figure 2D). No significant changes in cell viability were observed up to 60 nM, approximately double the concentration used in these studies.

To confirm that the effects of Co(III)-Ebox on *E-cadherin* promoter activity are a consequence of its ability to interfere with Snai1 DNA binding, we performed EMSAs on lysates from MDA-MB-231 cells that had been treated with Co(III)-Ebox for 24 h (Figure 2E). Treatment of MDA-MB-231 cells with 35 or 40 nM Co(III)-Ebox led to a significant reduction of DNA binding by endogenous Snai1, resulting in a reduction to 55.0% of the vector control at 35 nM and 25.4% at 40 nM Co(III)-Ebox, both of which are significantly different from the vector control (P = 0.025 for 35 nM and P = 0.0013 for 40 nM, Figure 2G). Although Co(III)-Ebox reduces Snai1 DNA binding, it does not alter total Snai1 protein levels (Figure 2E, Figure S6). Reduction of Snail DNA-binding in MDA-MB-231 cells was also achieved using a shRNA targeting *Snai1* (Fig. 2F). In this case, however, the decrease in DNA binding correlated with decreased Snail protein expression, confirming the distinct mechanisms employed by these two inhibitory agents (Fig. 2F).

### Neural crest formation is inhibited by Co(III)-Ebox in a temperature- dependent matter

Neural crest cells have proven to be a powerful in vivo model for investigating the role of Snail proteins in EMT and invasive cell behaviour, as well as the regulatory mechanisms that control the function of Snail factors in numerous developmental and pathological processes [36], [37]. We therefore examined the efficacy of Co(III)-Ebox for inhibiting Snail function during neural crest development in *Xenopus* embryos. This system provides an excellent in vivo model for determining the effectiveness of the Co(III)-Ebox conjugate when administered to complex tissues. In *Xenopus*, both *Snai1* and *Snai2* are expressed in pre-migratory neural crest precursor cells and contribute to the stem cell like properties of these cells [4], [7]. Loss of Snail function leads to loss of neural crest precursor cells, providing a powerful assay for the ability of Co(III)-Ebox to inhibit Snai1/2 function in vivo.

**Figure 3:**
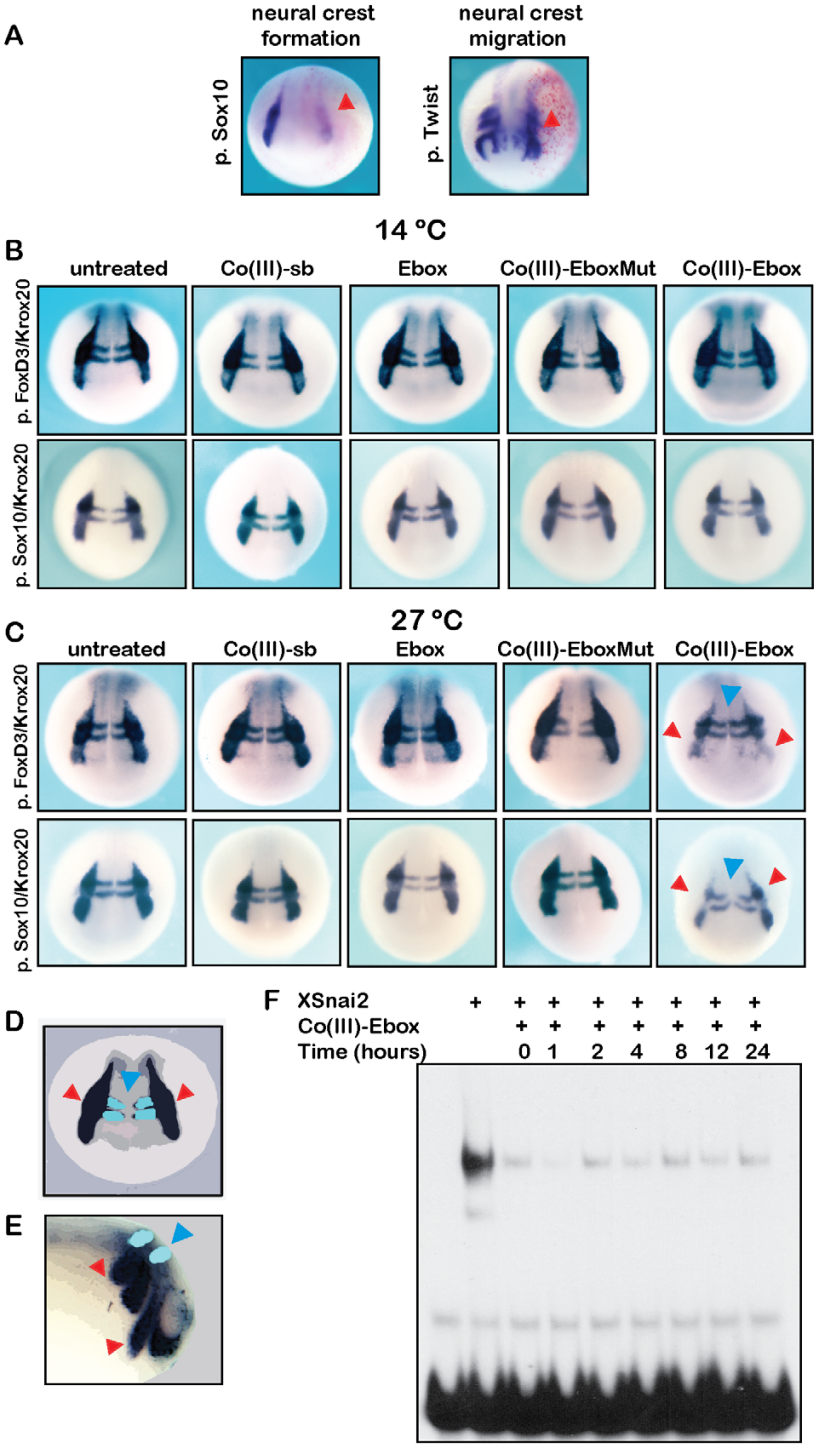
Co(III)-Ebox inhibits Snail-mediated neural crest cell specification in *Xenopus* via a temperature-dependent mechanism. (A) *In situ* hybridization of *Sox10* expression showing neural crest formation (left) and *Twist* expression showing neural crest migration (right) of embryos injected with Co(III)-Ebox into one cells of embryos at the 8-cell stage. Embryos were co-injected with β-galactosidase mRNA as a lineage tracer (red staining). Neural crest forming/migrating regions indicated with arrowheads on the injected side. (B, C) *In situ* hybridization showing the neural crest of control embryos or embryos injected with Co(III)-sb, Ebox, Co(III)-EboxMut or Co(III)-Ebox and grown at 14°C (B) or 27°C (C). Top panel: whole mount *in situ* hybridization showing *FoxD3* expression in the neural crest and *Krox20* expression in rhombomeres 3/5 of the CNS. For *FoxD3* embryos reared at 14°C, Co(III)-sb n = 12; Ebox n = 16; Co(III)-EboxMut n = 16; and Co(III)-Ebox n = 13. For *FoxD3* embryos reared at 27°C, Co(III)-sb = 13; Ebox n = 18; Co(III)-EboxMut n = 14; and Co(III)-Ebox n = 18. Bottom panel: expression of *Sox10* in the neural crest and *Krox20* (blue arrowheads). For *Sox10* embryos reared at 14°C, Co(III)-sb n = 21; Ebox n = 33; Co(III)-EboxMut n = 35; and Co(III)-Ebox n = 38. For *Sox10* embryos reared at 27°C, Co(III)-sb = 33; Ebox n = 35; Co(III)-EboxMut n = 35; and Co(III)-Ebox n = 36. Loss of *Sox10* and *FoxD3* is seen in the neural crest forming regions of embryos treated with Co(III)-Ebox grown at 27°C (red arrowheads). (D) Cartoon representation of expected *in situ* hybridization result for *Krox20* and neural crest markers (*Sox10* and *FoxD3*) at stage 17. Black staining represents neural crest forming regions visualized by *in situ* hybridization for *Sox10* or *FoxD3* and indicated by red arrows. Pale blue staining indicates hindbrain forming regions of rhombomeres 3 and 5 visualized by *in situ* hybridization with *Krox20* and indicated by blue arrows. (E) Cartoon representation of expected *in situ* hybridization result for *Krox20* and neural crest markers (*Sox10* and *Twist*) during neural crest cell migration. Black staining represents migrating neural crest regions visualized by *in situ* hybridization for *Sox10* or *Twist* and indicated by red arrows. Pale blue staining indicates hindbrain forming regions of rhombomeres 3 and 5 visualized by *in situ* hybridization with *Krox20* and indicated by blue arrows. (F) Co(III)-Ebox incubated in embryo lysate for 0 to 24 hours retains the and ability to inhibit XSnai2 DNA binding as visualized by EMSA.

To assess the consequence of Co(III)-Ebox on neural crest cell formation and migration, Co(III)-Ebox was injected, along with β-galactosidase mRNA as a lineage tracer, into single cells of eight-cell stage embryos. When injected embryos were examined by *in situ* hybridization at neurula stages, we found that *Sox10* expression was consistently diminished on the Co(III)-Ebox-injected side of the embryo (Figure 3A). At stages when neural crest cells have commenced migration, we found that cells on the Co(III)-Ebox injected side did not migrate to the same extent as those cells on the uninjected control side as visualized by *Twist* expression (Figure 3A). Importantly, however, we sometimes observed defects in neural crest cell formation/migration on the side of the embryos contra-lateral to the injection site, which is not observed following injection with cell autonomous agents such as mRNA.

Because of the early administration of Co(III)-Ebox in these experiments, the defects observed in neural crest cell migration could be a secondary consequence of depleting the neural crest cell population. Moreover, if Co(III)-Ebox can function non-cell autonomously and cross the midline, it could also inhibit Snail function in the mesoderm in addition to the neural crest. We therefore sought to identify protocols for administering Co(III)-Ebox that would allow greater spatial and temporal control over its activity.

To inhibit Snail at a developmental time more proximal to the formation of neural crest precursor cells, Co(III)-sb, Ebox, Co(III)-Ebox or Co(III)-EboxMut were introduced into the blastocoel of developing *Xenopus* embryos during late gastrula stages, when the process of neural crest induction is underway. Introduction of the inhibitory agent at this stage would avoid effects on Snail protein function in the mesoderm during earlier (blastula and early gastrula) stages. Injected embryos were cultured to neurula stages (stage 18/19), and the formation of neural crest precursor cells was evaluated using *in situ* hybridization for *FoxD3* and *Sox10*, expression of which marks the neural crest precursor population at these stages. In wildtype embryos, neural crest precursor cells border the neural plate/prospective CNS (see cartoon in Figure 3D). Embryos were simultaneously probed for expression of *Krox20*, which is expressed in two stripes of cells in the hindbrain CNS (pale blue cells marked by blue arrow in the Figure 3D). Hindbrain expression of *Krox20* is not dependent on Snai1/2 function, and therefore this gene serves as a normalization control. In addition, because some of our preliminary experiments had suggested that the ability of Co(III)-Ebox to inhibit target proteins might be at least partially temperature dependent, we examined *FoxD3* and *Sox10* expression in embryos that had been reared at either 14°C or 27°C following treatment with experimental or control compounds.

We found that when embryos were cultured at 14°C, they displayed normal expression of *FoxD3* or *Sox10* following treatment with either Co(III)-Ebox or control compounds (Figure 3B, Figure S7). By contrast, when embryos treated with Co(III)-Ebox were reared at 27°C, expression of both *FoxD3* and *Sox10* was substantially diminished, whereas expression of *Krox20* remained unchanged (Figure 3C, Figure S7). Importantly, treatment of embryos with Co(III)-sb, Ebox, or Co(III)-EboxMut had no effect on the expression of *FoxD3* or *Sox10* (Figure 3C, Figure S7). Together these findings indicate that Co(III)-Ebox inhibits Snail-dependent neural crest precursor formation in a temperature dependent manner.

For the Co(III)-Ebox conjugate to be of most utility as an experimental or therapeutic inhibitor of Snail protein function, it must be stable and retain its inhibitory activity over time. In order to examine the stability of Co(III)-Ebox with respect to its Snail inhibitory activity, cell lysates prepared from *Xenopus* embryos were treated with the compound. At designated time points, the continued ability of Co(III)-Ebox to inhibit Snail protein function was assayed by introducing a defined amount of in vitro translated XSnai2 protein to the Co(III)-Ebox treated embryo lysates. Following incubation in the conjugate-treated lysates, the DNA binding ability of the Snail protein was evaluated by EMSA. We found that the Co(III)-Ebox conjugate remained stable and active in the cellular lysates for at least 24 hours, as over this time period it retained the ability to efficiently inhibit DNA binding by XSnai2 (Figure 3F).

### Temperature-dependent inhibition of neural crest cell migration by Co(III)-Ebox inhibition of Snail

**Figure 4:**
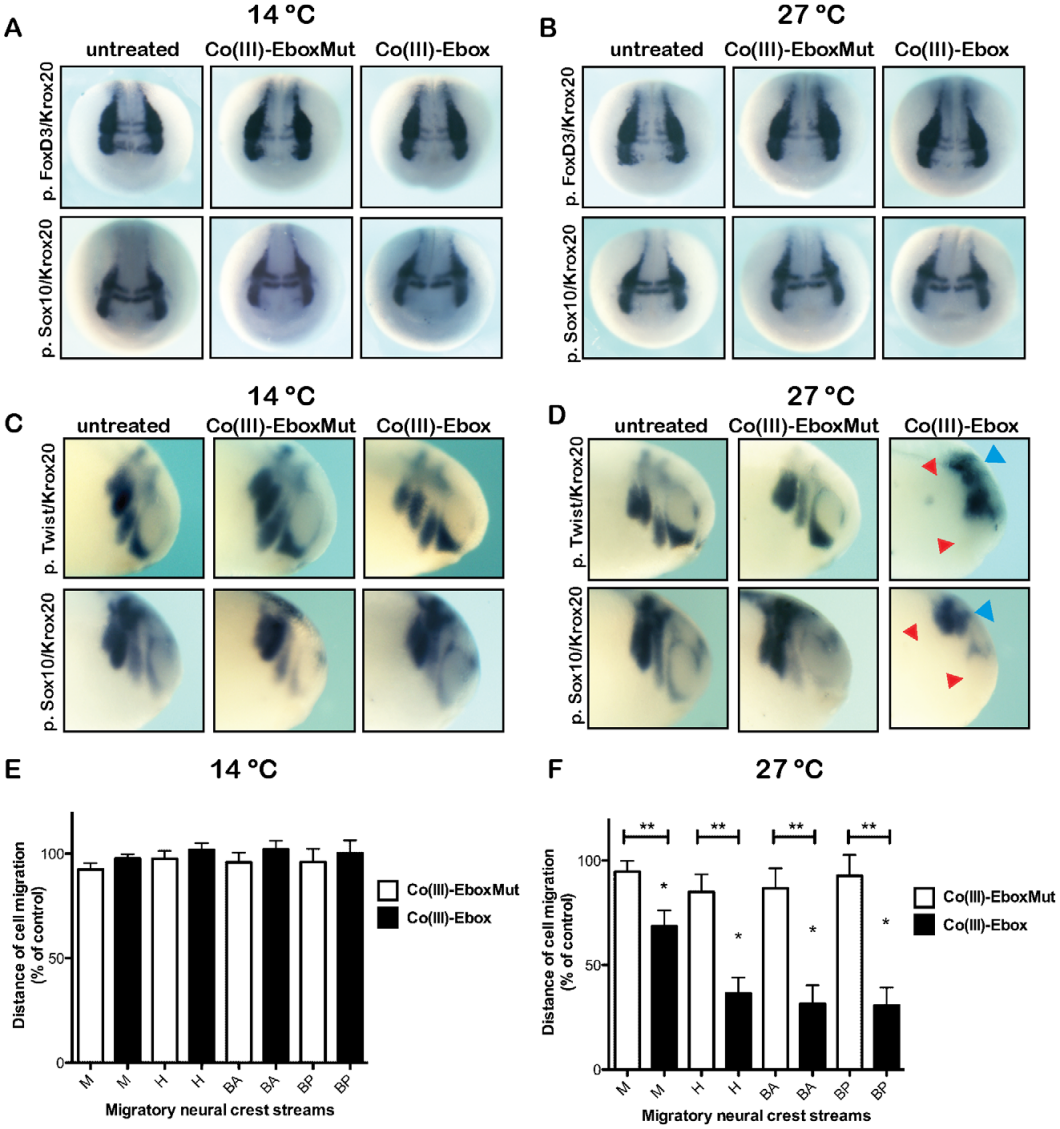
Co(III)-Ebox impairs neural crest cell migration in *Xenopus* embryos independently of neural crest specification. (A–D) *In situ* hybridization showing neural crest of embryos injected with Co(III)-Ebox or Co(III)-EboxMut during neurulation stages maintained at 14°C (A, C) or 27°C (B, D). (A, B) Co(III)-Ebox does not affect pre-migratory neural crest at 14°C (A) or 27°C (B) seen by *in situ* hybridization for *FoxD3* (top panel) or *Sox10* (bottom panel) For *FoxD3* embryos maintained at 14°C, untreated n = 14; Co(III)-EboxMut n = 15; and Co(III)-Ebox n = 15. For embryos maintained at 27°C, untreated = 15; Co(III)-EboxMut n = 15; and Co(III)-Ebox n = 15. For *Sox10* embryos maintained at 14°C, untreated n = 12; Co(III)-EboxMut n = 15; and Co(III)-Ebox n = 15. For embryos maintained at 27°C, untreated = 15; Co(III)-EboxMut n = 15; and Co(III)-Ebox n = 15. *Krox20* is used to visualize non neural crest tissue in the hindbrain. (C) Embryos treated with Co(III)-Ebox have normal neural crest migration at 14°C. (D) Embryos treated with Co(III)-Ebox grown at 27°C show impaired neural crest migration (red arrowheads) visualized by *in situ* hybridization for *Twist* and *Krox20* (top panel) or *Sox10* and *Krox20* (bottom panel). For *Twist* embryos maintained at 14°C, untreated n = 14; Co(III)-EboxMut n = 14; and Co(III)-Ebox n = 14. For embryos maintained at 27°C, untreated n = 11; Co(III)-EboxMut n = 10; and Co(III)-Ebox n = 17. For *Sox10* embryos maintained at 14°C, untreated n = 14; Co(III)-EboxMut n = 11; and Co(III)-Ebox n = 12. For embryos maintained at 27°C, untreated n = 13; Co(III)-EboxMut n = 12; and Co(III)-Ebox n = 14. (E, F) Quantification of normalized neural crest migration from *Twist* expression represented as means ± s.e.m. M = mandibular, H = hyoid, BA = anterior branchial, BP = posterior branchial. (E) T-tests determined no significant difference between embryos at 14°C. (F) T-tests determined that neural crest streams of embryos treated with Co(III)-Ebox are significantly different from controls [single star (*), M, P = 0.0002; H, P<0.0001; BA, P<0.0001 and BP, P<0.0001] and from embryos treated with Co(III)-EboxMut [double stars (**)] M, P = 0.0245; H, P = 0.0004, BA, P = 0.0005; and BP, P = 0.0001] at 27°C.

In addition to the essential role that Snail family transcription factors play in the formation of the neural crest stem cell population, they are also required later in neural crest cell development for the onset of migration. This role is linked to the ability of Snail proteins to mediate EMT and downregulate cell adhesion molecules such as E-cadherin, leading to delamination of neural crest cells from the neuroepithelium [38]. In order to determine if Co(III)-Ebox could specifically inhibit the effects of Snail on cell behavior, bypassing effects on the precursor cell population, Co(III)-Ebox or Co(III)-EboxMut was introduced into the archenteron at neurula stages (stage 13), after neural crest precursor cells had already formed. To ensure that treatment at these stages did not affect the precursor population, treated embryos were cultured at the restrictive (14 °C) or permissive (27 °) temperature to stage 18/19 (late premigratory stages) and examined by *in situ* hybridization for expression of *FoxD3* or *Sox10*. Expression of both of these genes was unchanged (Figure S8), indicating that the maintenance of the neural crest cell population was not impaired by Co(III)-Ebox or Co(III)-EboxMut when administered at these stages (Figure 4A and 4B). These results provide novel temporal insights into the requirements for Snail protein function during neural crest precursor formation, strongly suggesting that after their essential role in the initial establishment of the precursor population, Snail function is dispensable at least until the onset of neural crest migration.

To examine if Co(III)-Ebox disrupts neural crest cell migration when administered after neural crest precursor cells have formed, embryos treated with Co(III)-EboxMut or Co(III)-Ebox were allowed to develop to stage 25–28 when the cranial neural crest has migrated ventrally to the pharyngeal pouches. Neural crest cell migration was unimpaired in Co(III)-Ebox treated embryos and control embryos reared at 14 °C as assessed by the distances migrated by *Twist* and *Sox10* expressing neural crest cells (Figure 4C, cartoon in Figure 3E depicts normal pattern of neural crest migration marked by red arrows). *Krox20* expression in rhombomeres 3/5 of the hindbrain and in migratory neural crest cells was also normal (Figure 4C). Quantification of distance migrated by *Twist* expressing cells revealed no significant differences between untreated embryos or those treated with Co(III) or Co(III)-EboxMut (Figure 4E).

In marked contrast to embryos reared at 14 °C, when Co(III)-Ebox treated embryos were reared at 27 °C they displayed significant defects in neural crest migration. These embryos showed a decrease in distance migrated by all neural crest streams (mandibular, hyoid, anterior and posterior branchial) as compared to uninjected control embryos or Co(III)-EboxMut injected embryos, as assessed by *Twist*, *Sox10* or *Krox20* expression (Figure 4D). *Krox20* expression in the hindbrain was unimpaired in these experiments; expression in all treated embryos was indistinguishable from untreated embryos (Figure 4D). Similarly, embryos injected with Co(III)-EboxMut displayed normal neural crest cell migration as compared to uninjected control embryos. Quantification of the distance migrated by *Twist* expressing neural crest cells showed that embryos injected with Co(III)-Ebox displayed an up to 87% decrease in migration distance when cultured at 27°C (Figure 4F). All streams of neural crest cells showed significant deficits in migration compared to control embryos, or embryos treated with Co(III)-EboxMut. In embryos treated with Co(III)-EboxMut, neural crest migration was indistinguishable from that in control embryos (Figure 4F).

### Co(III)-Ebox prevents the formation of the neural crest derived melanocytes

**Figure 5:**
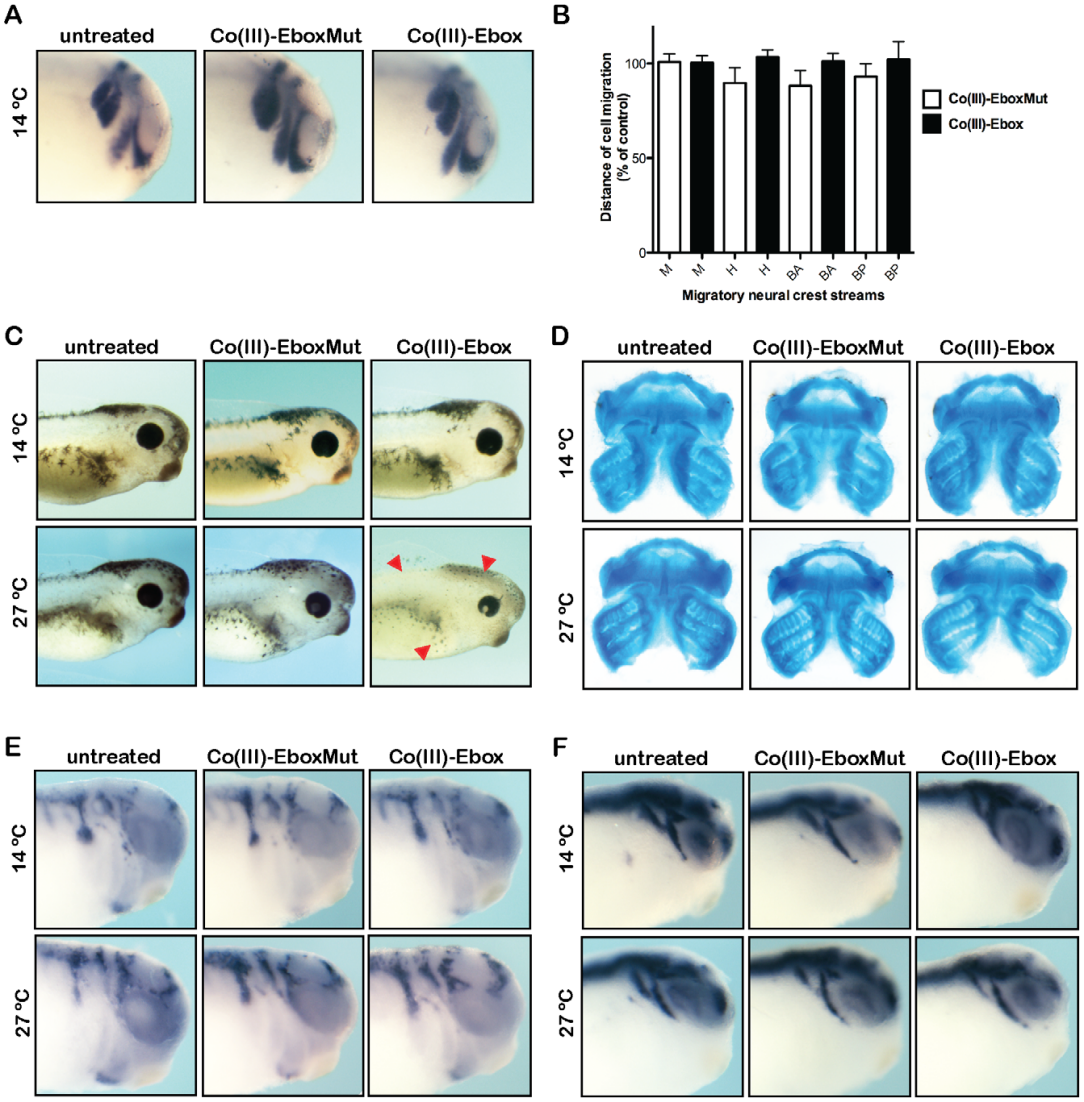
Temperature modulation of Co(III)-Ebox-mediated Snail inhibition reveals a requirement for Snail in melanocyte formation. Neurula stage embryos injected with Co(III)-Ebox or Co(III)-EboxMut in the archenteron space were maintained at 14°C and (A) neural crest migration visualized by whole mount *in situ* hybridization for *Twist* expression. *Krox20* is used to visualize rhombomeres 3/5 in the hindbrain. Untreated n = 8; Co(III)-EboxMut n = 8; and Co(III)-Ebox n = 12. (B) Quantification of mandibular, hyoid and branchial streams of neural crest cells are represented as the distance migrated as a percentage of control embryos. T-tests determined that neither Co(III)-EboxMut nor Co(III)-Ebox had a significant effect on neural crest cell migration. (C) Sibling embryos were maintained until swimming tadpole stages at 14 °C (top panel) or 27 °C (bottom panel) when melanocyte formation could be assessed. Arrowheads point to regions of diminished melanocytes. For embryos maintained at 14°C, untreated n = 12; Co(III)-EboxMut n = 12; and Co(III)-Ebox n = 12. For embryos maintained at 27°C, untreated n = 12; Co(III)-EboxMut n = 12; and Co(III)-Ebox n = 13. (D) Same embryos were maintained until swimming tadpole stages at 14 °C (top panel) or 27 °C (bottom panel) when craniofacial cartilage formation could be assessed. Cartliage was stained with Alcian blue and dissected to visualize cartilage formation. (E) Injected embryos were reared at 14 °C (top panel) or 27 °C (bottom panel) until stage 28 and glial cell formation was visualized by whole mount *in situ* hybridization for *FoxD3* expression. For embryos maintained at 14°C, untreated n = 15; Co(III)-EboxMut n = 17; and Co(III)-Ebox n = 11. For embryos maintained at 27°C, untreated n = 15; Co(III)-EboxMut n = 15; and Co(III)-Ebox n = 22. (F) Injected embryos were reared at 14 °C (top panel) or 27 °C (bottom panel) until stage 28 and primary neuron formation was visualized by whole mount *in situ* hybridization for *N-tubulin* expression. For embryos maintained at 14°C, untreated n = 15; Co(III)-EboxMut n = 17; and Co(III)-Ebox n = 13. For embryos maintained at 27°C, untreated n = 20; Co(III)-EboxMut n = 21; and Co(III)-Ebox n = 21.

Our findings demonstrate that Co(III)-Ebox can be used to distinguish temporally distinct requirements for Snail protein function during neural crest precursor formation and neural crest migration. We next sought to determine if we could use this inhibitor to identify additional, later, requirements for Snail proteins during neural crest cell fate diversification. For these experiments, Co(III)-Ebox or Co(III)-EboxMut was introduced into the archenteron at neurula stages (stage 13, as in Figure 4), and embryos were reared to neural crest migratory stages at 14°C. Under these conditions neural crest cell migration proceeded normally, as evidenced by unperturbed patterns of *Twist* and *Krox20* expression in migratory neural crest cells (Figure 5A). Quantification of the distance migrated by *Twist* expressing neural crest cells showed no significant differences regardless of treatment (Figure 5B). Once neural crest cell migration was underway, treated embryos were either maintained at 14°C or shifted to 27°C, and then cultured to stages when effects on neural crest cell fate diversification could be evaluated. Following normal neural crest cell migration along specific embryonic pathways, these cells differentiate into a diverse array of derivatives that includes neurons and glial cells of the peripheral nervous system, melanocytes, smooth muscle cells, connective tissue and craniofacial cartilage [39]. We therefore asked if Co(III)-Ebox-mediated Snail inhibition would interfere with the ability of neural crest cells to adopt any of these fates. Notably, we observed striking deficits in melanocyte formation in embryos that were treated with Co(III)-Ebox and reared at 27°C. By contrast, embryos treated with Co(III)-EboxMut exhibited normal numbers and patterns of melanocytes. Similarly, melanocyte formation was normal in embryos treated with Co(III)-Ebox or Co(III)-EboxMut and cultured at 14° C (Figure 5C, Figure S9).

The inhibition of melanocyte formation noted following Co(III)-Ebox-mediated Snail inhibition were in contrast for what we observed when we examined neural crest derived cranial cartilage, neurons, and glial cells. We found that craniofacial cartilage formation was normal in all treated embryos at both 14° C and 27° C (Figure 5D, Figure S9). Similarly, the formation of PNS neurons and glia in the cranial ganglia, as visualized by *N-tubulin* and *FoxD3* expression respectively, was unperturbed at stage 28 following Co(III)-Ebox-mediated Snail inhibition, or with control treatment (Figure 5E and 5F, Figure S9). These data suggest that in addition to playing required roles in the formation of neural crest precursor cells, and in the EMT/migratory behaviour of these cells, Snail plays a subsequent role in the formation of neural crest derived melanocytes. Our data also suggests that additional Snail function may not be required for the formation of cartilage, cranial neurons and glia, although it remains possible that the delivered dose was sufficient to block Snail function in melanocyte precursors but not in the precursors of neural crest derived cartilage, neurons and glia. The results in melanocytes, however, further highlight the utility of the Co(III)-Ebox conjugate as a potent chemical inhibitor of Snail family function that can be administered with high temporal resolution. The stability, potency, and temperature dependence of this agent make it a powerful tool for studying a family of transcriptional regulatory proteins that play multiple essential roles in embryonic development and are core mediators of both developmental and pathological EMTs. Our findings further suggest that this agent may hold therapeutic promise as an inhibitor of tumor metastasis.

## Discussion

Snail family zinc finger transcriptional repressors are essential for the formation of the neural crest stem cells, and are also required later in neural crest development for the onset of invasive and migratory behaviour. The proliferative, anti-apoptotic, migratory, and invasive properties that Snail factors confer on neural crest cells are recapitulated when *Snai1* and *Snai2* and other core regulators of EMT, including *Zeb1* and *Zeb2*, are expressed in epithelial tumor cells. The Snail protein family is highly studied for these reasons, and inhibitors of Snail function are therefore of high potential impact and importance for both basic science and clinical applications.

We have shown Snail factors can be potently inhibited by Co(III)-Ebox in vitro and in vivo. Inhibiting DNA binding by Snail proteins alleviates Snail-mediated transcriptional repression. The effects of Co(III)-Ebox are highly selective, as sequence specific controls with two base substitutions have greatly diminished effects on Snail function, strong evidence that off-target effects are limited. In vitro data further confirms that Co(III)-Ebox does not inhibit other Ebox-binding proteins that do not contain zinc finger domains, including MitF. Together these data strongly substantiate the specificity of Co(III)-Ebox for Ebox-binding zinc finger transcription factors, and indicate that there are at most nominal off-target effects on other zinc finger proteins, or other Ebox-binding proteins such as bHLH transcription factors.

In contrast to RNAi approaches, Co(III)-Ebox-mediated inhibition of Snai1 blocks Snai1 activity without affecting Snai1 protein levels. Co(III)-DNA conjugates therefore represent a novel approach for specifically blocking the function of targets in vitro and in vivo, without the requirement for decreasing the expression level of the protein itself. While in the present report we utilize this approach to block Snail protein function in tumor derived cells and early embryos, this methodology should prove applicable to targeted regulation of transcriptional events by a broad range of potential protein targets.

**Figure 6:**
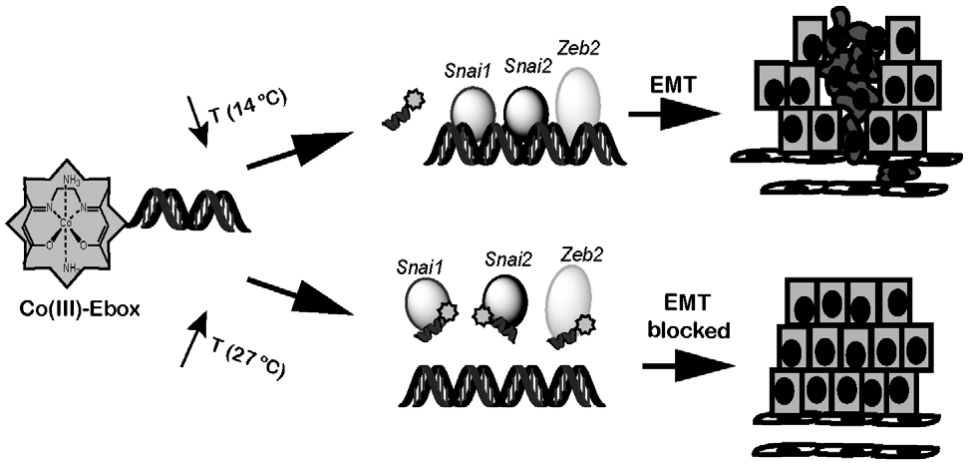
Proposed model for the temperature-dependent inactivation of proteins targeted by Co(III)-Ebox.

To demonstrate the utility of the Co(III)-Ebox conjugate, we have used it to probe the temporal requirements for Snail family function during neural crest development in *Xenopus*. We have used two approaches to controlling the timing of Co(III)-Ebox-mediated Snail inhibition in early embryos. Control over the time of application is a major advantage that chemical inhibitors hold over genetic means of inhibiting protein activity. Reagents such as RNAi affect the translation of new proteins but do nothing to inhibit protein already expressed within a cell, which must turn over before functional effects will be observed. Moreover, the temperature dependence of Co(III)-Ebox function affords an additional level of control that will prove of great value in model organism-based studies. As a consequence the Co(III)-DNA conjugate can be introduced into cells or tissues at one time but kept at a temperature where it is largely inactive. Because the reagent remains stable in the cytoplasm for long periods of time, Co(III)-Ebox-mediated inhibition can subsequently be activated by shifting to a higher temperature (Figure 6).

In this study we demonstrate the power that this level of control affords. We first demonstrate the specificity and temporal control of Co(III)-Ebox by showing that this means of inhibiting Snail function phenocopies the effects of molecular genetic approaches for inhibiting Snail function, while limiting off-target effects [1], [7], [9], [40]. Co(III)-Ebox effectively blocks the formation of the neural crest precursor cells and the subsequent migration of these cells, while not impairing the formation of the CNS or mesoderm. Indeed, the current experiments shed further light on the temporal requirements for Snail function at premigratory neural crest stages. These experiments indicate that once neural crest precursors have formed, Snail function is not required for the maintenance of these precursors, as indicated by the continued expression of neural crest markers even in the presence of Co(III)-Ebox. The next functional consequence of Co(III)-Ebox-mediated Snail inhibition was the failure of proper neural crest migration. To examine if there were further requirements for Snail function, such as during neural crest cell fate diversification, we activated Co(III)-Ebox only after neural crest migration was well underway. In this way we found that Snail function was required for the normal formation of at least one neural crest derivative, melanocytes, whereas neural crest derived cartilage and cranial neurons and glia developed normally despite inhibition of Snail function.

Snail repressors are known to directly or indirectly regulate the expression of other transcription factors in the complex gene regulatory network that controls neural crest formation and differentiation. In *Xenopus*, Snai1 is known to positively affect *Sox10* expression during the formation of neural crest precursor stem cells, presumably indirectly [41]. This is significant given that at later stages of development *Sox10* has been shown to play an essential role in the formation of the pigment cell lineage including melanocytes [42]. Although a role for Snail-dependent regulation of *Sox10* at these stages has not been previously reported, it is possible that inhibition of melanocyte formation by Co(III)-Ebox could result from the down-regulation of *Sox10* following inhibition of Snail function. *FoxD3* also plays roles in pigment cell and glia formation [43] and is positively regulated by Snail, however, we found that F*oxD3* expression in glial cells was unperturbed at stage 28 in treated embryos. Moreover, given that *Sox10* has been shown to promote both melanocyte and glial cell formation while inhibiting neuron formation [44], the normal development of glia and neurons in treated embryos suggests that *Sox10* function is not lost in all lineages. Further examination of the temporal requirements of Snail function, and its impact on the expression of other neural crest regulatory factors, should lead to a fuller understanding of the role of Snail in melanocyte formation as well as other neural crest cell derivatives.

Our findings on the effects of Co(III)-Ebox-mediated Snail inhibition in neural crest cells have implications beyond the development of this important cell type. Previous studies have reported the utility of Co(III) Schiff base complexes as targeted protein inhibitors in vitro [27], [28], [29], [30]. Moreover, non-targeted Co(III) Schiff base complexes have demonstrated some efficacy as viral inhibitors following topical application. [45], [46], [47]. Our findings on the inhibition of transcription factors in tumor-derived cells, and in neural crest stem cells, are the first report of a Co(III) Schiff base complex functioning as an intracellular and even nuclear inhibitor in vivo, and the first used in developmental studies, and as such represent a significant advancement in the field.

Our results further demonstrate the power and potential of Co(III)-DNA conjugates for the study of developmental events regulated by zinc finger transcription factors, and highlight the exceptional temporal control afforded by these agents. Generation of Co(III) Schiff base complexes targeted to other zinc finger transcription factors should prove a broadly applicable strategy for targeted inhibition of factors that play key roles in other developmental and disease processes. The flexible nature of such DNA-conjugated Co(III) Schiff base complexes permits more facile development of inhibitors compared to conventional small molecule drugs, since novel inhibitors can be rationally designed simply by changing the targeting sequence. In the case of Co(III)-Ebox, our findings suggest that this conjugate holds significant therapeutic promise as an inhibitor of Snail-dependent tumor progression and recurrence. We further show that this agent can be used in model organisms to shed important light on the function of this important family of transcriptional regulatory proteins.

## Materials and Methods

### Plasmids and Snai1 shRNA

Luciferase reporter gene constructs containing wild-type *E-cadherin* promoter sequences were a gift from E. Fearon [35]. The *E-cadherin* promoter region containing 3 Eboxes, from −108 to +125 of the endogenous *E-cadherin* gene, was cloned into pGL2-Basic upstream of firefly Luciferase (Ecad-luc). Ebox elements in the *E-cadherin* promoter in Ecad-luc were mutated from 5′-CAGGTG-3′ to 5′AAGGTA-3′ in EcadMut-luc. Murine *Snail* (MSnail) cDNA was cloned into a pCS2 variant that adds 5 myc tags to the N- terminus as has been previously described [30]. A short hairpin RNA (shRNA) targeting *Snail* in pLKO was obtained from Sigma Aldrich (St. Louis, MO).

### Reporter gene assays

Cell lines were transfected using ExGen500 (Fermentas) according to manufacturer's protocol. In experiments examining repression of the *E-cadherin* reporter gene construct by endogenous Snail, 200 ng *E-cadherin* construct (Ecad-luc or EcadMut-luc), 50 ng of Renilla luciferase construct as a control and the indicated amount of experimental compound [Ebox, Co(III)-sb, Co(III)-EboxMut, or Co(III)-Ebox] were transfected per well. To determine the effects of Co(III)-Ebox and other experimental compounds on MSnail repression of the *E-cadherin* promoter, 20 ng of *MSnail* was transfected with 200 ng *E-cadherin* construct, 50 ng of Renilla luciferase construct as a control and the indicated amount of experimental compound [Ebox, Co(III)-sb, Co(III)-EboxMut, or Co(III)-Ebox]. In each experiment the total DNA transfected in each well was equalized by the addition of non-coding DNA as pCS2+ empty vector to samples that do not contain experimental compound.

Cell extracts were prepared 24 h after transfection using Passive lysis buffer (Promega Corp.) followed by determination of firefly luciferase and Renilla luciferase activity using the Dual-Luciferase Reporter assay kit (Promega Corp). Results were normalized by dividing by Renilla activity and are reported as fold inductions. Statistical analysis was performed on means using a t-test.

### Electrophoretic mobility shift assay

Electrophoretic mobility shift assays for XSnai2 and XMitF were performed as previously described [27]. Images presented are a representative replicate of triplicate samples. Band intensities were quantified on a STORM 680 PhosphorImager (GE Healthcare, Piscataway, NJ). Slug-bound band intensity values were normalized to the background signal in each individual lane using ImageQuant 5.2. The percentage of residual complex bound to Slug probe is the normalized intensity of each treatment divided by the normalized intensity of the untreated lane. These values for Co(III)-Ebox were averaged over three replicates and reported with the standard error. Statistical analysis was performed on the means using a t-test.

### Co(III)-Ebox stability in embryo lysates

Untreated *X. laevis* embryos were collected and lysed at the blastula stage. Co(III)-Ebox (0.1 µM) was incubated at room temperature in embryo lysate for 0, 1, 2, 4, 8, 12 and 24 hours. At each of the indicated times an aliquot of Co(III)-Ebox in embryo lysate was removed and frozen at −80 °C. Once all of the timepoints had been collected and frozen *X. laevis* Snai2 (Slug) protein in vitro translated from plasmid DNA using the TNT® Reticulocyte lysate system (Promega Corp.) was added to each sample along with EMSA binding buffer and ^32^P-labeled Slug probe, and samples were separated by electrophoretic mobility shift assay as previously described [27].

### Western Blot Analysis

For Western blots, cell lysates from EMSA were used. Samples were denatured and resolved on SDS/PAGE. Proteins were detected using polyclonal Snail1-specific rabbit polyclonal antibody was obtained from Abcam (Cambridge, MA) and polyclonal Actin-specific rabbit antibody was obtained from Sigma-Aldrich (St. Louis, MO). Immunoreactive bands were detected using an enhanced chemiluminescence solution (SuperSignal West Pico Chemiluminescent Substrate; Pierce, Rockford, IL). Band images were obtained by using ChemiDoc XRS+ (Bio-Rad, Hercules, CA) and band intensity analyzed by Image Lab™ software version 2.0.1 (Bio-Rad, Hercules, CA). Images presented are a representative replicate of triplicate samples. Snai1 band intensity values were normalized to the Actin signal in each individual lane. The percentage of residual Snai1 expressed is the normalized intensity of each treatment divided by the normalized intensity of the untreated lane. These values for were averaged over three replicates and reported with the standard error. Statistical analysis was performed on the means using a t-test.

### Embryo preparation and methods

Pigmented and albino eggs from *Xenopus laevis* were obtained and fertilized using standard protocols [48]. All embryos are staged using the Niewkoop-Faber method. Embryos were microinjected into one cell at the 8-cell stage, the blastocoel at stage 10 or the archenteron space at stage 13 using a micromanipulator. Whole mount *in situ* hybridization was performed with digoxigenin-labeled antisense RNA probes as previously described [49].

The distance of neural crest cell migration was measured in ImageJ 1.42I. The length of each embryo head was measured from the dorsal midline to the ventral side directly behind the eye cup. Each of the streams of neural crest cell migration was measured independently. The relative percentage of distance migrated of the neural crest of Co(III)-Ebox or Co(III)-EboxMut treated embryos was calculated by comparing the distance migrated of treated embryos to the distance migrated of untreated embryos.

For cartilage staining, embryos were fixed in formaldehyde at stages 40–43 and stained overnight in 0.2% alcian blue/30% acetic acid in ethanol. Embryos were washed through a glycerol series into 80% glycerol/2% KOH before manual dissection of cartilages.

All animal work was conducted according to relevant national and international guidelines and was approved by Northwestern University's Animal Care and Use Committee (approved protocol number 2010–0125, Animal Assurance Number A3283-01).

## Supporting Information

Figure S1 **Breast cancer cell lines express different levels of Snai1 protein.** Western Blot of Snai1 and Actin protein in a panel of breast cancer cell lines.
(TIF)

Figure S2 **Exogenous MSnail represses** ***E-cadherin*** **reporter gene activity in MCF7 cells.** *E-cadherin* reporter gene activity in MCF7 cells transfected with empty vector (pCS2+) or MSnai1. Values are expressed as relative light units compared to pCS2+ samples. Data are represented as means ± s.e.m., n = 4.
(TIFF)

Figure S3 **Co(III)-Ebox represses XSnai2 DNA-binding more effectively than Co(III)-EboxMut.** Lysates of uninjected blastula stage embryos (lane 1) or embryos with overexpressed XSnai2 protein (lanes 2–8) were incubated with increasing concentrations of Co(III)-Ebox (lanes 3–5) or Co(III)-EboxMut (lanes 6–8) of 0.5, 1, and 2 µM for 15 min before challenge with a ^32^P-labeled Ebox containing Slug DNA probe for 30 min. Samples were analyzed by EMSA on a native TBE/acrylamide gel. Multiple shifted complexes were observed for XSnai2 as previously reported [27], [50]. Co(III)-Ebox inhibits all specific complexes as seen in lanes 3–5 [27].
(TIF)

Figure S4 **Co(III)-Ebox represses XSnai2 DNA binding but does not affect XMitF DNA binding.** (A) Lysates of uninjected blastula stage embryos (lanes 1 and 4) or embryos with overexpressed XSnai2 protein (lanes 2 and 3) or XMitF (lanes 5–10) were incubated with increasing concentrations of Co(III)-Ebox of 0, 5, 15, 50, 150 or 500 nM for 15 min before challenge with a ^32^P-labeled Ebox containing Slug DNA probe for 30 min. Samples were analyzed by EMSA on a native TBE/acrylamide gel. (B) Western Blot analysis of XSnai2 and XMitF protein expression levels in embryo lysates using an antibody against Myc, as both XSnai2 and XMitF contain a 6× Myc epitope tag. Dilutions of mRNA were used to establish equalized protein levels for EMSA. Lysates from embryos injected with a 1∶200 dilution of XMitF mRNA or 1∶100 dilution of XSnai2 mRNA were used to examine DNA binding. Shifted bands for binding of XSnai2 (as in Figure S3), XMitF and a non-specific band for the MitF probe are indicated.
(TIF)

Figure S5 **Ebox-binding proteins including Snail factors expressed in MDA-MB-231 cells repress** ***E-cadherin*** **promoter activity.** Luciferase reporter gene activity of Ecad-luc and EcadMut-luc in MDA-MB-231 cells. The mutations in the Eboxes in EcadMut-luc do not allow for Ebox protein binding and effective transcriptional repression. Values are expressed as relative light units compared to Ecad-luc samples. Data are represented as means ± s.e.m., n = 4.
(TIFF)

Figure S6 Snai1 protein expression level in MDA-MB-231 cells after 24 h with 0 to 40 nM Co(III)-Ebox. Band intensities of Western blots of Snai1 were obtained by using ChemiDoc XRS+ analyzed by Image Lab™ software version 2.0.1. Snai1 band intensity values were normalized to the Actin signal in each individual lane. The percentage of residual Snai1 expressed is the normalized intensity of each treatment divided by the normalized intensity of the control. Data are represented as the means ± s.e.m., n = 3. Statistical analysis by t-test determined there was no significant difference between any of the means.
(TIFF)

Figure S7 Co(III)-Ebox inhibits Snail-mediated neural crest cell specification in *Xenopus* via a temperature-dependent mechanism. Graphical representation of the percentage of embryos exhibiting loss of neural crest cell formation as seen by *in situ* hybridization for (A) *FoxD3* or (B) *Sox10* of control embryos or embryos injected with Co(III)-sb, Ebox, Co(III)-EboxMut or Co(III)-Ebox and grown at 14°C or 27°C.
(TIF)

Figure S8 Co(III)-Ebox impairs neural crest cell migration in *Xenopus* embryos independently of neural crest specification. Graphical representation of the percentage of embryos exhibiting loss of neural crest cell formation as seen by *in situ* hybridization for (A) *FoxD3* or (B) *Sox10* of control embryos or embryos injected with Co(III)-sb, Ebox, Co(III)-EboxMut or Co(III)-Ebox and grown at 14°C or 27°C.
(TIF)

Figure S9 **Temperature modulation of Co(III)-Ebox-mediated Snail inhibition reveals a requirement for Snail in melanocyte formation.** Graphical representation of the percentage of embryos exhibiting loss of (A) melanocyte formation, (B) cranial cartilage formation, (C) glial cell formation (as seen by *in situ* hybridization for *FoxD3*), and (D) neuron formation as seen by *in situ* hybridization for *N-tubulitn* of control embryos or embryos injected with Co(III)-EboxMut or Co(III)-Ebox and grown at 14°C or 27°C.
(TIF)
