# Supplementary figures and images for: Targeted Inactivation of Snail Family EMT Regulatory Factors by a Co(III)-Ebox Conjugate
Source: PLoS One. 2012 Feb 29;7(2):e32318. doi: 10.1371/journal.pone.0032318 (PMC3290632; doi:10.1371/journal.pone.0032318)

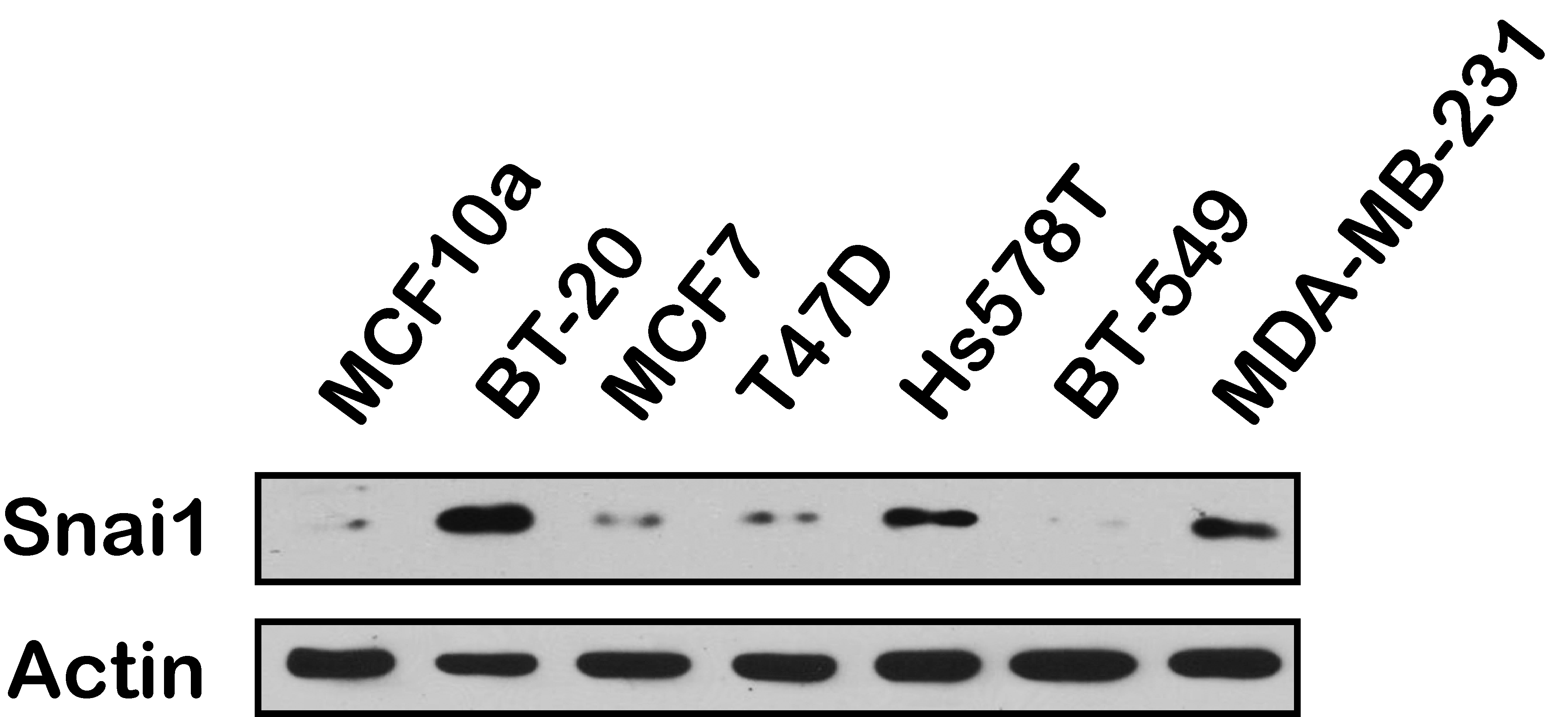

Supplement: Figure S1 — Breast cancer cell lines express different levels of Snai1 protein. Western Blot of Snai1 and Actin protein in a panel of breast cancer cell lines. (TIF) [file pone.0032318.s001.tif]

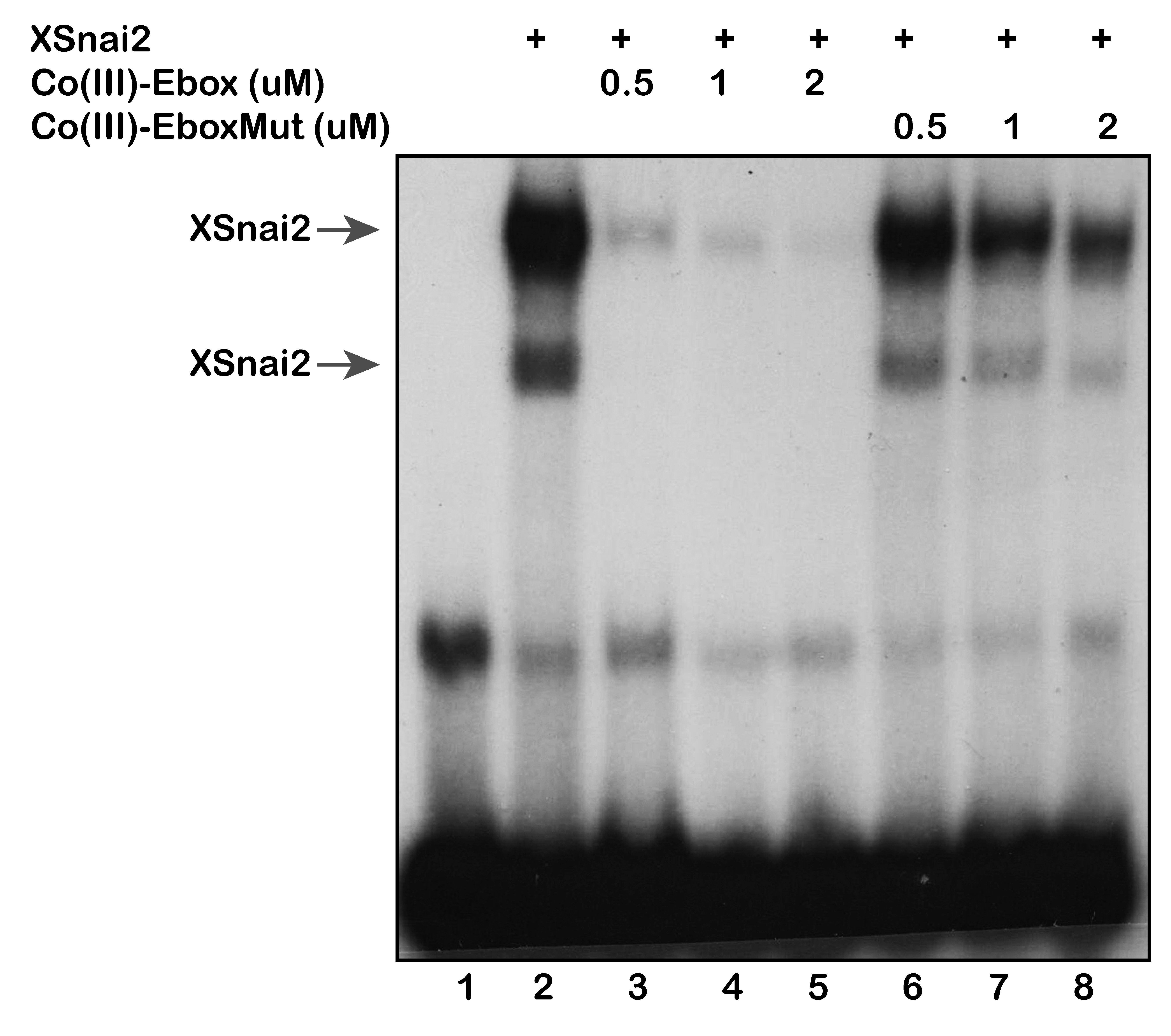

Supplement: Figure S3 — Co(III)-Ebox represses XSnai2 DNA-binding more effectively than Co(III)-EboxMut. Lysates of uninjected blastula stage embryos (lane 1) or embryos with overexpressed XSnai2 protein (lanes 2–8) were incubated with increasing concentrations of Co(III)-Ebox (lanes 3–5) or Co(III)-EboxMut (lanes 6–8) of 0.5, 1, and 2 µM for 15 min before challenge with a 32P-labeled Ebox containing Slug DNA probe for 30 min. Samples were analyzed by EMSA on a native TBE/acrylamide gel. Multiple shifted complexes were observed for XSnai2 as previously reported [27], [50]. Co(III)-Ebox inhibits all specific complexes as seen in lanes 3–5 [27]. (TIF) [file pone.0032318.s003.tif]

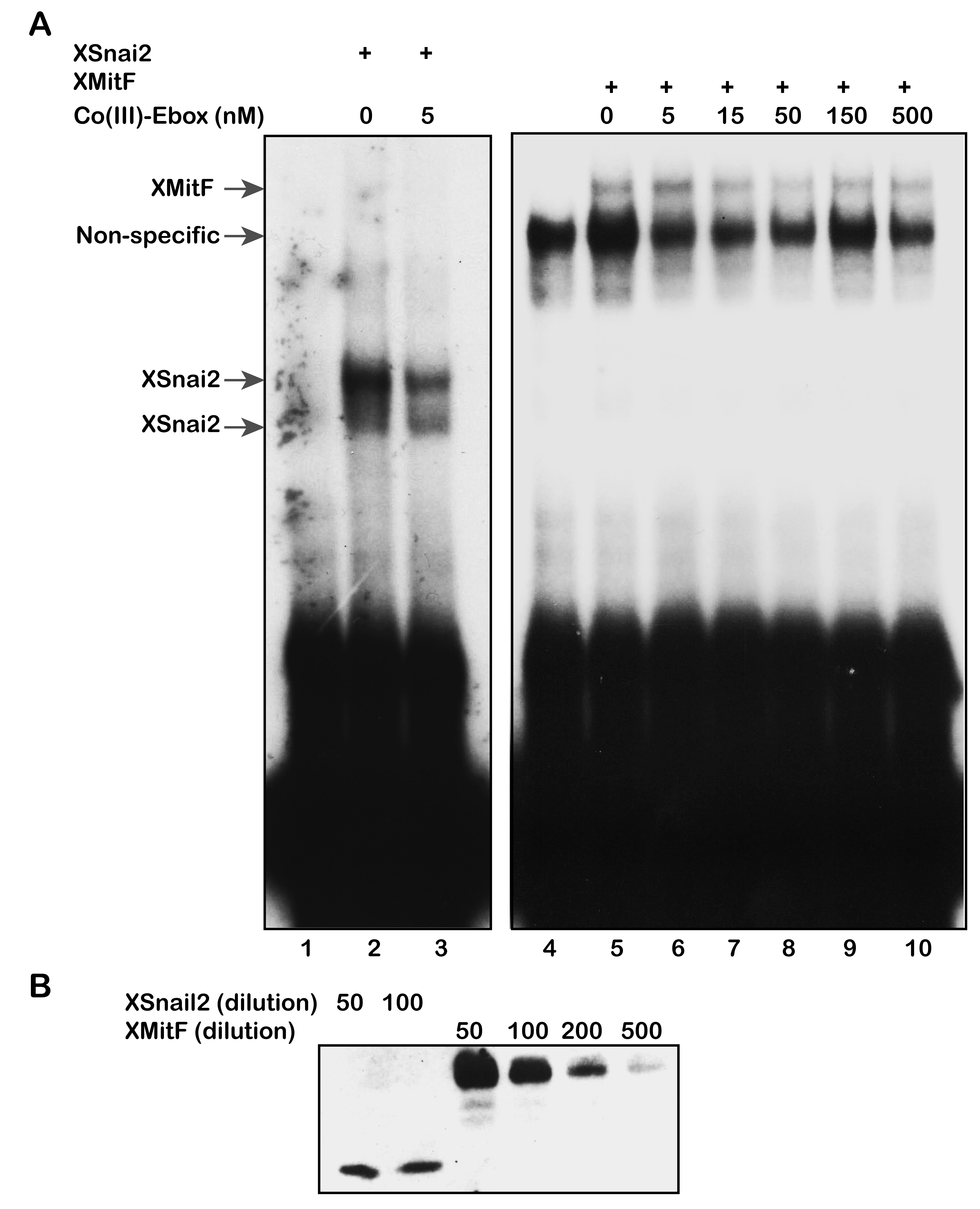

Supplement: Figure S4 — Co(III)-Ebox represses XSnai2 DNA binding but does not affect XMitF DNA binding. (A) Lysates of uninjected blastula stage embryos (lanes 1 and 4) or embryos with overexpressed XSnai2 protein (lanes 2 and 3) or XMitF (lanes 5–10) were incubated with increasing concentrations of Co(III)-Ebox of 0, 5, 15, 50, 150 or 500 nM for 15 min before challenge with a 32P-labeled Ebox containing Slug DNA probe for 30 min. Samples were analyzed by EMSA on a native TBE/acrylamide gel. (B) Western Blot analysis of XSnai2 and XMitF protein expression levels in embryo lysates using an antibody against Myc, as both XSnai2 and XMitF contain a 6× Myc epitope tag. Dilutions of mRNA were used to establish equalized protein levels for EMSA. Lysates from embryos injected with a 1∶200 dilution of XMitF mRNA or 1∶100 dilution of XSnai2 mRNA were used to examine DNA binding. Shifted bands for binding of XSnai2 (as in Figure S3), XMitF and a non-specific band for the MitF probe are indicated. (TIF) [file pone.0032318.s004.tif]

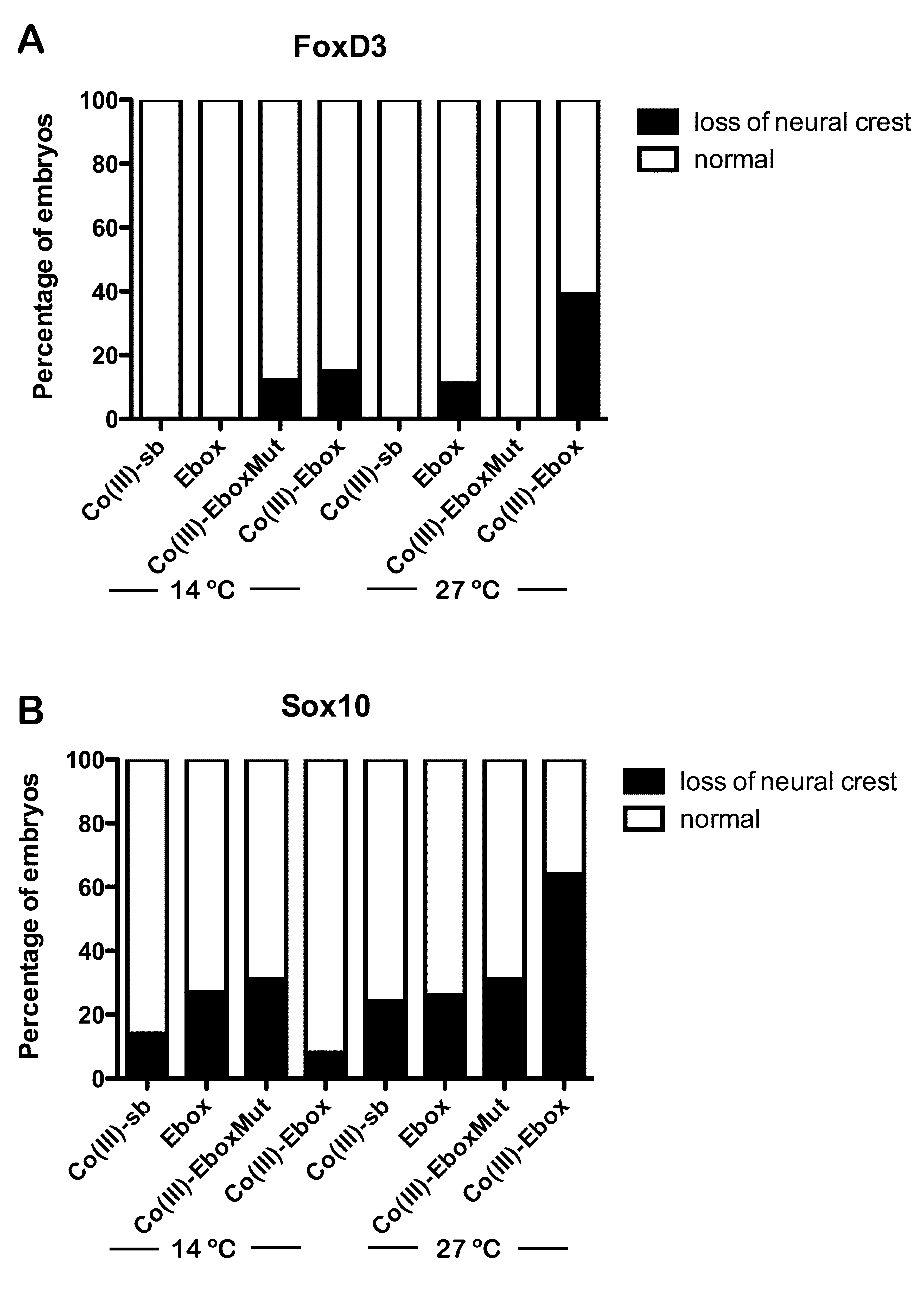

Supplement: Figure S7 — Co(III)-Ebox inhibits Snail-mediated neural crest cell specification in Xenopus via a temperature-dependent mechanism. Graphical representation of the percentage of embryos exhibiting loss of neural crest cell formation as seen by in situ hybridization for (A) FoxD3 or (B) Sox10 of control embryos or embryos injected with Co(III)-sb, Ebox, Co(III)-EboxMut or Co(III)-Ebox and grown at 14°C or 27°C. (TIF) [file pone.0032318.s007.tif]

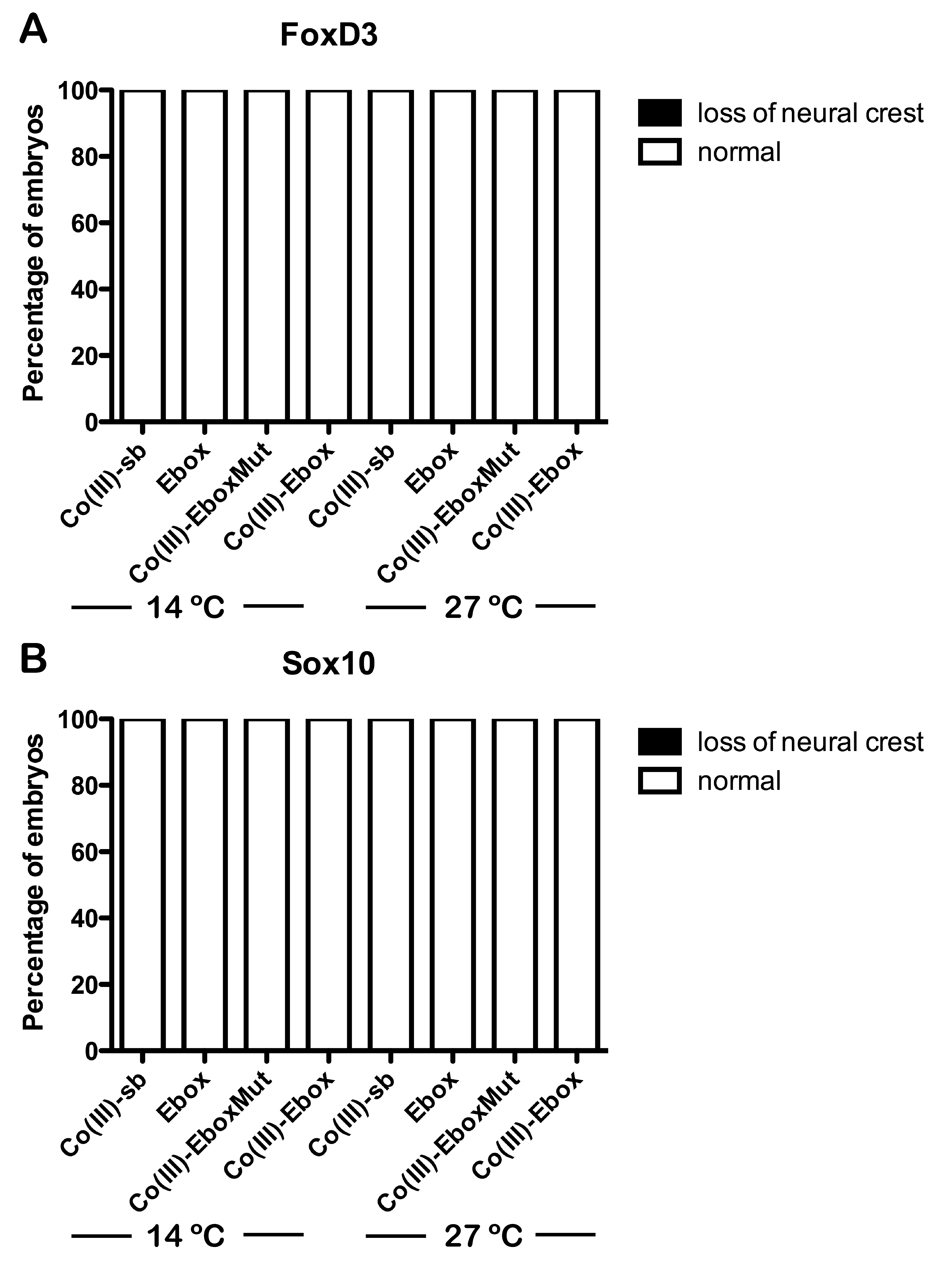

Supplement: Figure S8 — Co(III)-Ebox impairs neural crest cell migration in Xenopus embryos independently of neural crest specification. Graphical representation of the percentage of embryos exhibiting loss of neural crest cell formation as seen by in situ hybridization for (A) FoxD3 or (B) Sox10 of control embryos or embryos injected with Co(III)-sb, Ebox, Co(III)-EboxMut or Co(III)-Ebox and grown at 14°C or 27°C. (TIF) [file pone.0032318.s008.tif]

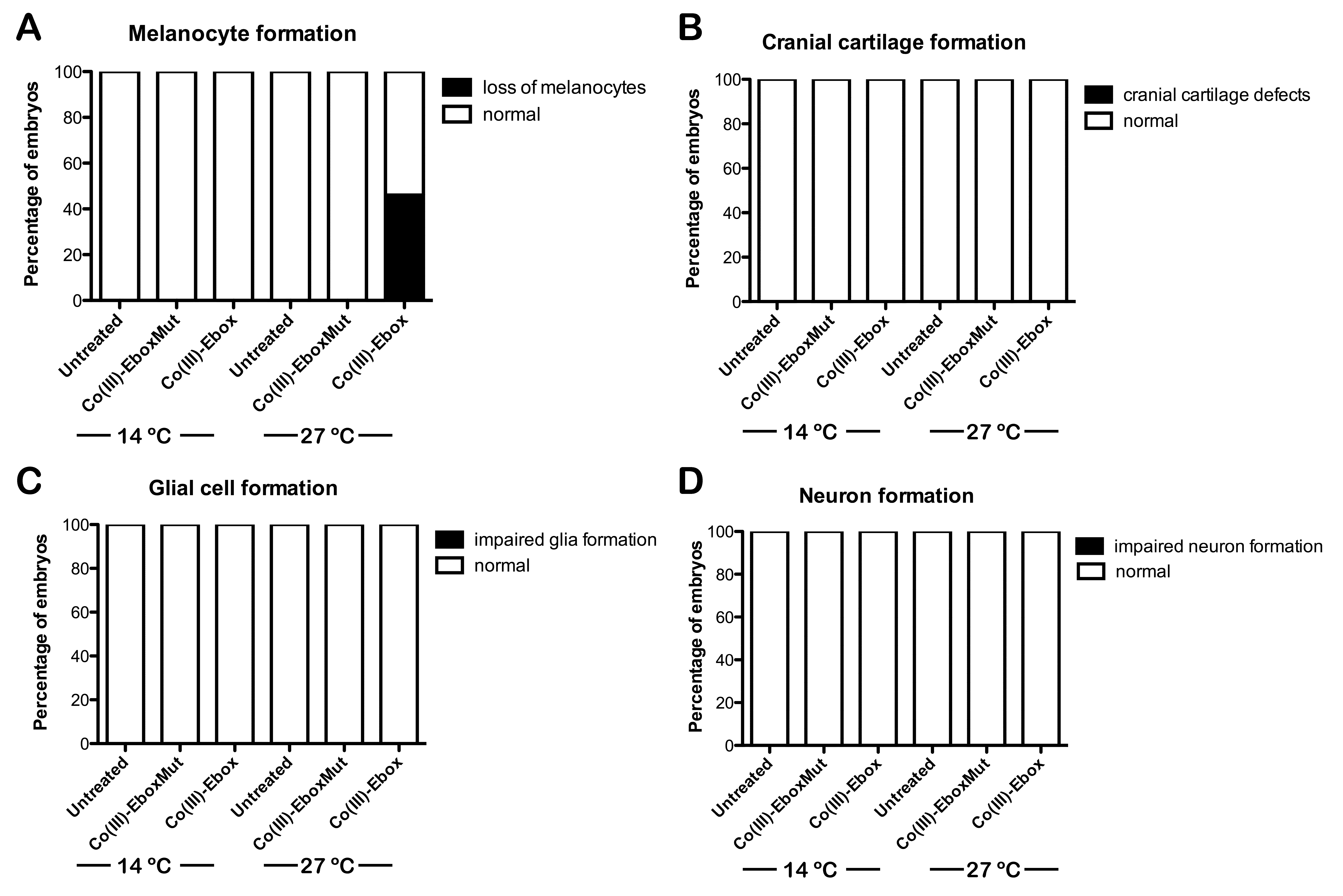

Supplement: Figure S9 — Temperature modulation of Co(III)-Ebox-mediated Snail inhibition reveals a requirement for Snail in melanocyte formation. Graphical representation of the percentage of embryos exhibiting loss of (A) melanocyte formation, (B) cranial cartilage formation, (C) glial cell formation (as seen by in situ hybridization for FoxD3), and (D) neuron formation as seen by in situ hybridization for N-tubulitn of control embryos or embryos injected with Co(III)-EboxMut or Co(III)-Ebox and grown at 14°C or 27°C. (TIF) [file pone.0032318.s009.tif]
